# Macro-level determinants of nature connectedness: An exploratory analysis of 61 countries

**DOI:** 10.1007/s13280-025-02275-w

**Published:** 2025-11-01

**Authors:** Miles Richardson, Michael Lengieza, Mathew P. White, Ulrich S. Tran, Martin Voracek, Stefan Stieger, Viren Swami

**Affiliations:** 1 School of Psychology, University of Derby, Kedleston Road, Derby, DE22 1GB UK; 2 Department of Psychology, Durham University, South Road, Durham, DH1 3LE UK; 3 Department of Clinical and Health Psychology, Faculty of Psychology, University of Vienna, Kolingasse 14/16, 1090 Vienna, Austria; 4 Department of Cognition, Emotion, and Methods in Psychology, Faculty of Psychology, University of Vienna, Vienna, Austria; 5 Department of Psychology and Psychodynamics, Karl Landsteiner University of Health Sciences, Krems an Der Donau, Austria; 6 School of Psychology, Sport, and Sensory Sciences, Anglia Ruskin University, Cambridge, UK; 7 Centre for Psychological Medicine, Perdana University, Kuala Lumpur, Malaysia

**Keywords:** Human–nature relationship, Indicators, Metrics, Nature connectedness, Sustainability

## Abstract

Nature connectedness is increasingly recognised as a causal issue in environmental crises and a powerful strategy for transformative change. However, little is known about how it varies across countries and the macro-level factors that influence the human–nature relationship at a societal level. Using a large dataset (*N* = 56 968) from a diverse set of 61 countries, this study explored how both objective country-level indicators of the socio-ecological context and subjective country-level indicators of socio-political values were related to nature connectedness. Using linear, factor, and network analysis, several objective (urbanicity and business environment) and subjective country indicators (scientific and religious values) were significantly associated with nature connectedness. These and other factors are combined into a proposed conceptual model of key macro-factors in the human–nature relationship that can inform future research and policy initiatives.

## Introduction

Nature connectedness is a well-established construct that reflects an individual’s cognitive and emotional connection to nature (Mayer and Frantz 2004; Tam 2013). Often measured as the perceived overlap between self and nature (Schultz 2002) or a sense of oneness with nature (Mayer and Frantz 2004), higher levels of nature connectedness are associated with improved human wellbeing (Pritchard et al. 2020; White et al. 2021) and greater pro-environmental behaviours and positive attitudes towards nature (Barragan-Jason et al. 2022; Mackay and Schmitt 2019). Conversely, lower levels of nature connectedness are recognised as one of three major underlying causes of biodiversity loss (IPBES 2024). Consequently, promoting stronger nature connectedness is likely to be a powerful strategy for the transformative change required to address the environmental crises (IPBES 2024). This growing recognition of nature connectedness as a transformative lever raises important questions about its societal-level determinants.

### Knowledge gaps in nature connectedness research

Targeting nature connectedness to transform the human–nature relationship has become an important focus for policy proposals (e.g. IPBES 2024; SEI and CEEW 2022). However, little is known about how nature connectedness varies across countries, as well as the macro-level factors that influence it at a societal level. A better understanding of such macro-level factors can inform the design and delivery of initiatives at deeper leverage points to enhance nature connectedness at the scale and pace necessitated by the global crises, while accounting for regional variations.

Extant nature connectedness research has focussed on individual-level factors. Using a variety of valid and reliable psychometric scales (c.f., Tam 2013), research has identified various factors that shape, and are affected by, nature connectedness. For example, the higher levels of nature connectedness that can be fostered through engaging with and noticing nature can lead to higher levels of pro-nature conservation behaviours (Pocock et al. 2023). However, much less is known about macro-level antecedental factors that may shape individual nature connectedness at a societal scale. An initial step towards overturning this neglect was provided by Richardson et al. (2022). They first proposed several country-level indicators, which they grouped into four broad dimensions of nature connectedness (Kellert 1993; Lumber et al. 2017). An exploratory analysis of the relationship between these metrics and nature connectedness with data from 14 European countries (*N* = 14 745) showed that consumption and commerce (e.g. affluence and technology use) were significantly associated with lower levels of nature connectedness (Richardson et al. 2022). These findings highlight the need to explore broader macro-level influences on nature connectedness, particularly those that go beyond objective socio-ecological indicators.

### Extending the scope: Towards a global perspective

A recent study involving a wider range of 41 countries, albeit with a relatively small number of university environmental students per country (*N* = 4262) showed that nature connectedness varied across countries and was negatively related to higher levels of urbanisation, wealth, and development (Kleespies and Dierkes 2023). Although informative, the findings of both studies are unlikely to generalise to more diverse global populations. To address this gap, the present study extends previous work by incorporating both objective and subjective country-level indicators across a more diverse set of community-based samples from 61 countries, thereby enabling a more comprehensive understanding of the macro-level determinants of nature connectedness. These country-level indicators will now be introduced.

#### Socio-ecological context: Objective country-level indicators

The history of socio-ecological progress is generally thought to have influenced a disconnection from nature (Eckersley 2000; Hamilton 2002). Given the lack of data on how observable factors—such as consumption patterns—are related to nature connectedness, their inclusion can be supported by turning to studies on individual-level nature connectedness. For example, orientations towards materialism (Hedlund-de Witt et al. 2014) and consumerism (Mayer and Frantz 2004) are negatively correlated with nature connectedness. Other research indicates that a greater individual orientation towards dominionistic tendencies is also negatively correlated with nature connectedness (Ng and Leung 2022). Factors that reflect a negativistic human–nature relationship (e.g. risks from nature or poor weather; Brown 2017; Duffy and Verges 2010; Zsido et al. 2022) are similarly associated with lower levels of individual nature connectedness. Lastly, the decline of nature experiences—due to factors such as loss of biodiversity (Samus et al. 2022), urbanisation, land use (Balázsi et al. 2019), and technology (Larson et al. 2019)—are frequently identified as a predictor of individual nature connectedness (see Lengieza and Swim 2021). These four broad socio-ecological dimensions (i.e. consumption, dominion, negativistic, and nature experience) have objective metrics. Those used by Richardson et al. (2022) were included in the present paper with minor adjustments to account for the data available across a much wider range of countries (see Table 2 for details).

#### Socio-political values: Subjective country-level indicators

Relationships with nature are likely shaped by both objective ‘external’ influences (e.g. consumption, dominion, negativistic, and nature experience) and also ‘internal’ values (e.g. worldviews, beliefs, and attitudes; see Lengieza and Swim 2021, for a review on the impact of worldviews on nature connectedness). Consequently, in addition to the objective factors that may shape nature connectedness, this section will explain why we investigate the use of four dimensions of culturally shared social values and attitudes as potential macro-factors.

*Social Values and Attitudes:* Several social values and attitudes may impact nature connectedness. Social values—such as valuing interpersonal relationships, pro-sociality, and trust—could be related to nature connectedness due to the link between pro-social behaviours and nature connectedness (Lengieza et al. 2023a, b). Technological development and usage have also been associated with nature connectedness (e.g. Kesebir and Kesebir 2017), suggesting that attitudes towards technology could be a factor. Additionally, the stability of a nation’s society and whether a population is settled or facing crises might influence an individual’s relationship with nature.

*Economy versus the Environment*: The balance between economic growth and environmental protection provides values and attitudes related to nature connectedness (Lengieza and Swim 2021). Environmental values are also indicated by the level of membership in environmental organisations, which demonstrates pro-environmental attitudes—an additional predictor of nature connectedness (see Lengieza and Swim 2021). Lastly, economic values encompass equality and individualism, factors that influence the human–nature relationship (Logan and Prescott 2022).

*Religious Values:* Religion is recognised as important in understanding the social values that shape the human–nature relationship (Ives and Kidwell 2019), whether positively (Brown 2017; Hedlund-de Witt et al. 2014) or negatively (Vess et al. 2012). Additionally, there has been increased attention on spirituality and religion in understanding the values influencing the human–nature relationship (Ives and Kidwell 2019). For example, Indigenous peoples frequently maintain a strong spiritual connection with nature that is formed through cultural practices (Niigaaniin and MacNeill 2022; Gauthier et al. 2025). Thus, religious values likely impact nature connectedness.

*Political Culture:* Freedom and democracy are topics discussed in the broader human–nature connectedness literature (e.g. Chandler 2013). Various studies have suggested that individual-level nature connectedness may be associated with different political views. For instance, Neumayer (2004) indicated a positive association with left-leaning political views. In contrast, Drescher et al. (2022) found indications of a stronger relationship to nature connectedness among politically right-leaning land-owners.

## Research objectives

The present study had two ancillary objectives. First, we sought to use the analysis of the objective and subjective factors to propose a tentative conceptual model of key macro-factors in the human–nature relationship and approaches to change. Second, we sought to test how nature connectedness compared to the Sustainable Development Goals Index as an indicator of the human–nature relationship.

In summary, this study explores the macro-level determinants of nature connectedness by addressing three interlocking research questions. First, it evaluates how well the socio-ecological indicator groupings conform to the 4-dimensional framework proposed by Richardson et al. (2022), thereby assessing the need for a new framework or conceptual model. Second, it examines which objective country-level socio-ecological indicators and subjective measures of socio-political values are most closely associated with nature connectedness. Third, those results are used to propose a new conceptual model. Finally, this study compares nature connectedness with the Sustainable Development Goals Index to determine its relative efficacy as a holistic indicator.

## Materials and methods

The present study made use of the Body Image in Nature Survey (BINS) dataset, which was collected via a researcher-crowdsourced project involving 253 scientists collaborating across 65 countries between November 2020 and February 2022 (Swami et al. 2022). The dataset consists of 56 968 respondents from 65 countries. Men comprised 40.5% (*n* = 23 083), 58.9% (*n* = 33 539) were women, and 0.6% (*n* = 346) were of another gender identity. Participants ranged in age from 18 to 99 years (*M* = 33.10, *SD* = 13.79). Most participants were single (42.0%, *n* = 23 955), 33.5% (*n* = 19 056) were married, 19.5% (*n* = 11 083) were in a long-term relationship but not married, and 5.0% (*n* = 2874) had another status.

### Approach

To identify macro-level factors associated with nature connectedness, ecological studies using aggregated socioeconomic data from geographical regions can help uncover previously unidentified factors in social conditions. This method is regarded as the initial step to generate population-level hypotheses (Loney and Nagelkerke 2014; Roumeliotis et al. 2021) and is particularly appropriate given the limited knowledge about the macro-level factors influencing the human–nature relationship at a societal scale. Nevertheless, it is crucial not to assume that statistical relationships at the group level necessarily apply to individuals within that group, especially when certain individuals may not be affected by the outcome under investigation (Loney and Nagelkerke 2014; Roumeliotis et al. 2021). In this instance, nature connectedness pertains to all individuals and no individual-level conclusions are being drawn. This ecological study benefits from addressing the ‘individualistic fallacy,’ which occurs when it is presumed that individuals are unaffected by their living environment (Loney and Nagelkerke 2014; Roumeliotis et al. 2021). Individual data supporting proposed macro-level associations, such as the link between biodiversity and individual nature connectedness, enhance the approach and facilitate drawing individual conclusions (Loney and Nagelkerke 2014). Therefore, to answer the research questions below, country-level indicators have been substantiated with reference to individual-level data where feasible.

(RQ1) How well do the socio-ecological context indicator groupings fit the four dimensions proposed by Richardson et al. (2022)?

(RQ2) Which objective country-level indicators of the socio-ecological context are associated with nature connectedness?

(RQ3) Which subjective country-level indicators of socio-political values are associated with nature connectedness?

### Measures

#### Nature connectedness

The BINS survey included the 14-item version of the well-validated Connectedness to Nature Scale (CNS-7; Mayer and Frantz 2004). However, a unidimensional model of the CNS with all 14 items had poor fit to the data (Swami et al. 2024), which led to the retention of a 7-item version of the CNS (i.e. CNS-7), which evidenced partial scalar invariance across all nations and languages represented in the BINS and full scalar invariance across age groups and gender identities. Nevertheless, composite reliabilities were below acceptable levels for data from two nations (Bosnia and Herzegovina and Iraq), so these data were excluded from analyses (Swami et al. 2024), leaving CNS-7 data from 63 nations. Table 1 displays the latent means of nature connectedness for these 63 countries, as reported by Swami et al. (2024). The majority of indicators were unavailable for Palestine and Taiwan, so data from these nations were also excluded from the present analyses, leaving data from 61 nations. For analyses involving biodiversity and mean income, countries with missing data on either variable were excluded using listwise deletion. This resulted in six countries being omitted from those specific analyses, while they were retained in analyses where complete data were available.

**Table 1:** Latent means of nature connectedness (after Swami et al. 2024), with the United Kingdom used as the anchor nation

Nepal	1.386
Iran	1.215
South Africa	1.200
Bangladesh	1.144
Nigeria	1.111
Chile	0.961
Croatia	0.944
Ghana	0.917
Bulgaria	0.883
Tunisia	0.862
Brazil	0.855
Palestine	0.837
Argentina	0.834
Latvia	0.827
Serbia	0.786
Philippines	0.781
Colombia	0.772
Taiwan	0.759
France	0.704
Malaysia	0.683
Malta	0.661
Turkey	0.655
Egypt	0.639
Slovenia	0.594
Estonia	0.591
Ecuador	0.518
Greece	0.516
Lithuania	0.507
Bahrain	0.488
India	0.466
Slovakia	0.456
Indonesia	0.442
Cyprus	0.439
Hungary	0.436
Kazakhstan	0.433
China	0.413
Thailand	0.413
Czechia	0.412
Portugal	0.369
Romania	0.347
Austria	0.330
Pakistan	0.323
UAE	0.310
Italy	0.280
Poland	0.279
Australia	0.248
USA	0.240
Lebanon	0.218
Iceland (English)	0.189
Ukraine	0.169
Norway	0.159
Switzerland	0.148
South Korea	0.142
Russia	0.094
Ireland	0.090
Saudi Arabia	0.078
United Kingdom	0.000
Netherlands	− 0.054
Canada (English)	− 0.067
Germany	− 0.080
Israel	− 0.303
Japan	− 0.391
Spain	− 0.613

#### Socio-ecological context: Objective country-level indicators

The metrics for the four dimensions are adapted from Richardson et al. (2022) and presented in Table 2.

**Table 2:** Objective country-level indicators

Metric	Expected dimension	Description	Source
Urban Population	Extinction of Experience	Percentage of total population living in an urban area. From United Nations Population division	World Bank Urbanization Prospects: 2018 Revision
Older Adults	Extinction of Experience	Percentage of the population aged 65 + years old	World Bank Population Prospects and age/sex distributions of United Nations Population Division: 2020 Revision
Biodiversity	Extinction of Experience	National Biodiversity Index. Values range between 1.0 (maximum: Indonesia) and 0.0 (Greenland)	National Biodiversity Index
Ease of Doing Business	Consumption and Commerce	Business friendly regulations	World Bank, Doing Business project
Mean Income	Consumption and Commerce	Survey mean consumption or income per capita, total population	World Bank, Global Database of Shared Prosperity
Technology	Consumption and Commerce	Individuals using the Internet (% of population)	World Bank
Energy Use	Consumption and Commerce	kWh per capita	World Bank from IEA Statistics
Cultivated Land	Utility and dominion	Percentage of arable and pasture)	Calculated from the CIA World Factbook-Land Use and CIA World Factbook-Area
Material Footprint	Utility and dominion	A consumption-based indicator of resource use	Column ‘O’ from Weidman et al. 2013
Natural disasters	Negativistic	Vulnerability to natural disaster	World Risk Index. Calculated – Exposure Column p. 56
Rainfall	Negativistic	Average precipitation in depth (mm per year)	Food and Agriculture Organisation
SDG Ranking	Comparison Item	Score on Sustainable Development Goals Index	SDG Index

#### Socio-political metrics: Subjective country-level indicators

The socio-political metrics—which serve as indicators of social, economic, environmental, religious, and political values—were derived from the World Values Survey (WVS), which provides data for numerous countries. Eighteen indicators (see Table 3 for details) potentially associated with nature connectedness were selected across the four dimensions of values introduced above. This selection aligns with the objective of ecological studies to identify factors and generate population-level hypotheses (Roumeliotis et al. 2021). These indicators are introduced according to their respective WVS categories.

**Table 3:** Subjective country-level indicators. Agreement with statements is related to higher scores

Item	WVS category	Question
Belief in Heaven	*Religious Values*	Which, if any, of the following do you believe in? Heaven
Importance of God	*Religious Values*	How important is God in your life?
Importance of Religion	*Social Values and Attitudes*	For each of the following, indicate how important it is in your life: Religion
Importance of Friends	*Social Values and Attitudes*	For each of the following, indicate how important it is in your life: Friends
Society needs radical change	*Social Values and Attitudes*	The entire way our society is organised must be radically changed by revolutionary action
Society needs gradual change	*Social Values and Attitudes*	Our society must be gradually improved by reforms
Society needs defending	*Social Values and Attitudes*	Our present society must be valiantly defended against all subversive forces
More Technology	*Social Values and Attitudes*	Please tell me for each one, if it were to happen, whether you think it would be a good thing, a bad thing, or don’t you mind? More emphasis on the development of technology
Income Equality	*Economic Values*	How would you place your views on this scale? Incomes should be made more equal. There should be greater incentives for individual effort
Environment over growth	*Economic Values*	Which of them comes closer to your own point of view? Protecting the environment should be given priority, even if it causes slower economic growth and some loss of jobs
Growth over Environment	*Economic Values*	Economic growth and creating jobs should be the top priority, even if the environment suffers to some extent
Trust in Neighbours	*Trust and Organisation Membership*	Could you tell me for each whether you trust people from this group completely, somewhat, not very much or not at all? Your neighbourhood
Environmental Membership	*Trust and Organisation Membership*	Could you tell me whether you are an active member, an inactive member or not a member of that type of organisation? Environmental organisation
Technology is good	*Science and Technology*	Science and technology are making our lives healthier, easier, and more comfortable
Science versus Faith	*Science and Technology*	We depend too much on science and not enough on faith
Left or Right	*Political Culture*	In political matters, people talk of ‘the left’ and ‘the right.’ How would you place your views on this scale, generally speaking? Note: Higher more right
Democracy	*Political Culture*	How important is it for you to live in a country that is governed democratically?
Freedom of Choice	*Political Culture*	Indicate how much freedom of choice and control you feel you have over the way your life turns out
Freedom vs. Equality	*Security*	Most people consider both freedom and equality to be important, but if you had to choose between them, which one would you consider more important?

### Analytical approach

For RQ1, to determine the appropriate number of factors to retain, Velicer’s minimum average partial (MAP) test (O’Conner 2000; Velicer 1976) and factor analysis were employed to provide insight into how well the selected indicators fit the framework based on the available data, taking into account the small sample size. Both exploratory factor analysis and linear analysis have been utilised in previous studies that consider country-level factors in an outcome variable (Schiavone et al. 2021; Vaitla et al. 2017). Due to the limited sample size, the exploratory factor analysis was conducted using the unweighted least squares method of extraction, as this method tends to better recover the factor structure (Jung 2013), with direct oblimin oblique rotation being applied because the variables are not orthogonal (Beauducel and Hilger 2023). Furthermore, a simple network analysis was performed to complement the factor analysis to visualise clusters and inter-relationships (Lin et al. 2022). The ties in the network represent the significant partial correlations (Kim 2015) between variables, with negative correlations depicted with red bands and positive correlations with green bands. Clusters were determined by a clustering algorithm (*Cluster_optimal()* from the igraph package; Csardi 2013) using the absolute value of the partial correlation and are indicated by the text colour. Correlations that were not significant were given a value of zero. We then used thresholding to remove trivial (*r* < 0.10) associations from the network, following established procedures (see Lengieza et al. 2025).

Research Questions 2 and 3 suggest using correlation and linear analysis. Linear analysis with regression is useful in both predictive and explanatory research and explains phenomena based on variables chosen by a theoretical framework (Pedhazur 1997). Recent studies indicate that as few as two cases per variable may suffice for linear analysis (Austin and Steyerberg 2015). Network analyses were conducted using the package igraph (Csardi 2013) in R (R Core Team 2024). Partial correlations were derived from *pcor()* in the *ppcor* package (Kim 2015). SPSS was used for all other analyses.

#### Variable reduction and selection

With low cases per variable, correlation-based variable selection should be based on statistical significance and relevance to the research context, including the indicator grouping framework. Therefore, variable selection for the linear analysis was based on significant (*p* < 0.05) correlations to nature connectedness and high inter-item correlations (*c*. 0.9).

#### Development of conceptual model: Analytical approach

To develop a meaningful and interpretable conceptual model of macro-level influences on nature connectedness, a mixed-method analytical strategy was employed. This approach integrated statistical factor analysis, network analysis, and theoretical interpretation grounded in Richardson et al.’s (2022) socio-ecological framework. The analytical process employed factor analysis and a network analysis to visualise inter-variable relationships and clustering. The model development was further informed by linear analysis, which identified significant predictors of nature connectedness from both objective socio-ecological indicators and subjective socio-political values.

## Results

*(RQ1) How well do the previously derived socio-ecological context indicator groupings fit the categories derived by Richardson et al’s (* 2022 *) typology?*

**Fig. 1:**
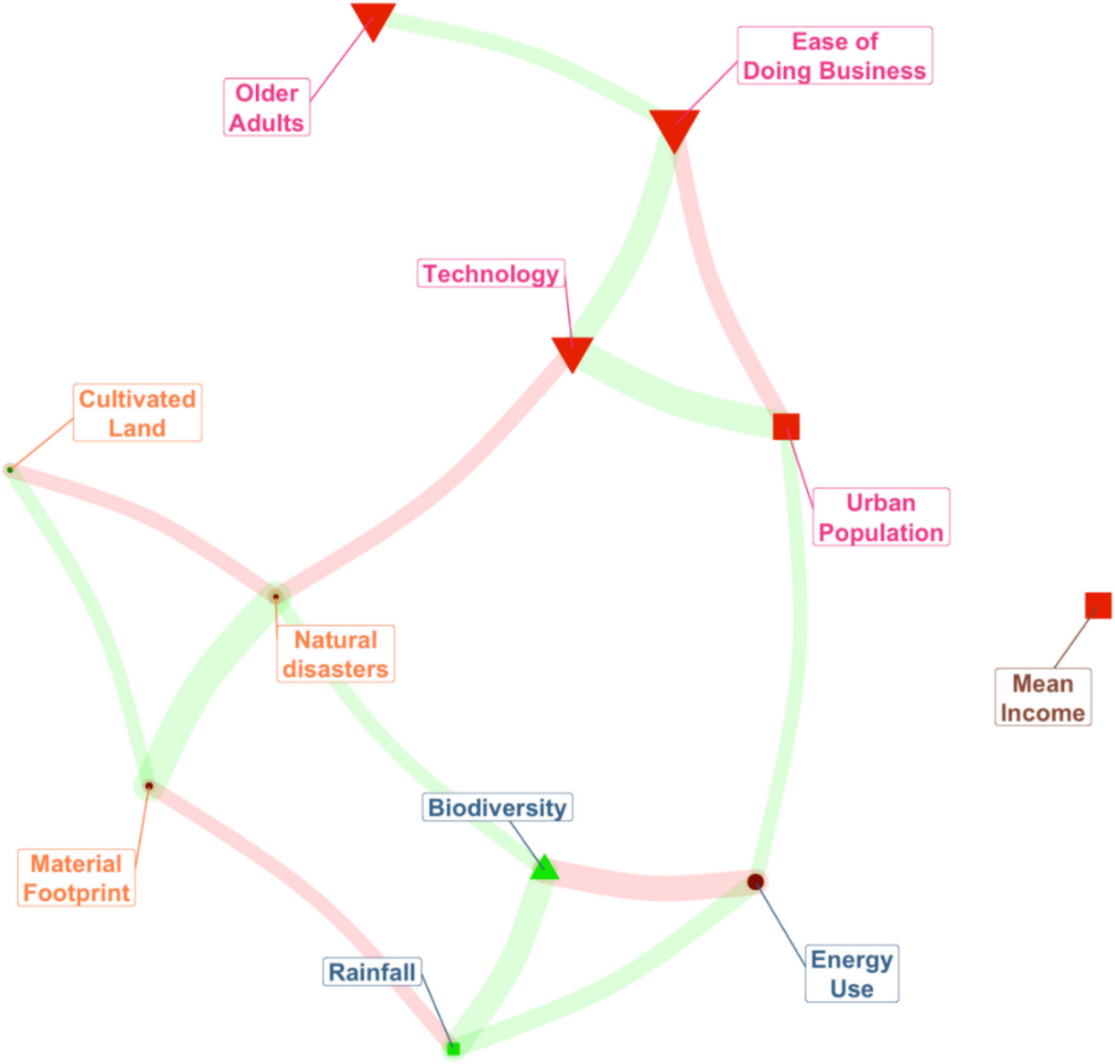
Network analysis of socio-ecological factors and their relationship to nature connectedness (Red nodes indicate a negative correlation with nature connectedness, as shown in the correlation matrix. Green nodes denote a positive correlation with nature connectedness. Triangles represent correlations in the top third for their colour group; squares for the middle third; circles for the bottom third. Red bands indicate negative partial correlations, while green bands indicate positive partial correlations)

The network analysis nodes illustrate the raw correlations between the selected variables and nature connectedness (see Fig. 1). The cluster grouped Older Adults, Ease of Business, Technology, and Urban Population together, as well as Material Footprint and Natural Disasters. Biodiversity, with its weaker factor loading, is clustered with rainfall.

In the factor analysis, the Kaiser (1960) criterion identified three distinct factors, which together explained 55% of the variation in the data. However, Velicer’s minimum average partial (MAP) test suggested a single factor might be more appropriate. As discussed further in the development of the tentative conceptual model, although Velicer’s MAP suggested a single underlying factor, a three-factor solution was chosen for its greater clarity and usefulness in guiding future research and policy. This approach preserves important distinctions between the three-factor solution, which would be lost in a single undifferentiated grouping, and is presented in Table 4. Table 4 shows that Factor 1 (Socioeconomic) contained six broadly socioeconomic items related to the consumption, commerce, and extinction of experience indicator groupings. Factor 2 (Environment) contained three items from three indicator groupings, but all were broadly environmental. Factor 3 (Weather and Land Use) contained rainfall with cultivated land having a weaker and negative factor loading. This 3-factor model offers a conceptually coherent framework that aligns with disciplinary boundaries and is supported by complementary network analysis findings. Inter-relationships between the factors were very small (correlations ranged from 0.04 to below 0.16), and items did not significantly overlap across factors (cross-loadings were negligible to small), suggesting that each factor captures a distinct theme.

**Table 4:** Pattern factor loadings and communalities for exploratory factor analysis of nature connectedness. Factor loadings > 0.4 in bold

	Factor 1	Factor 2	Factor 3	Communality
Urban Population	**0.631**	0.051	0.071	0.393
Older Adults	**0.576**	0.022	− 0.208	0.382
Mean Income	**0.723**	− 0.026	0.122	0.536
Ease of Business	**0.782**	0.153	− 0.094	0.611
Internet	**0.861**	− 0.088	− 0.061	0.783
Energy Use	**0.597**	− 0.081	0.183	0.399
Biodiversity	− 0.384	**0.446**	0.356	0.572
Material Footprint	0.148	**0.882**	− 0.226	0.769
Natural disasters	− 0.037	**0.860**	0.245	0.858
Rainfall	− 0.154	0.032	**0.697**	0.537
Cultivated Land	− 0.239	0.066	**− 0.418**	0.224

*RQ2: Which objective country-level indicators of socio-ecological context are associated with nature connectedness?*

**Fig. 2:**
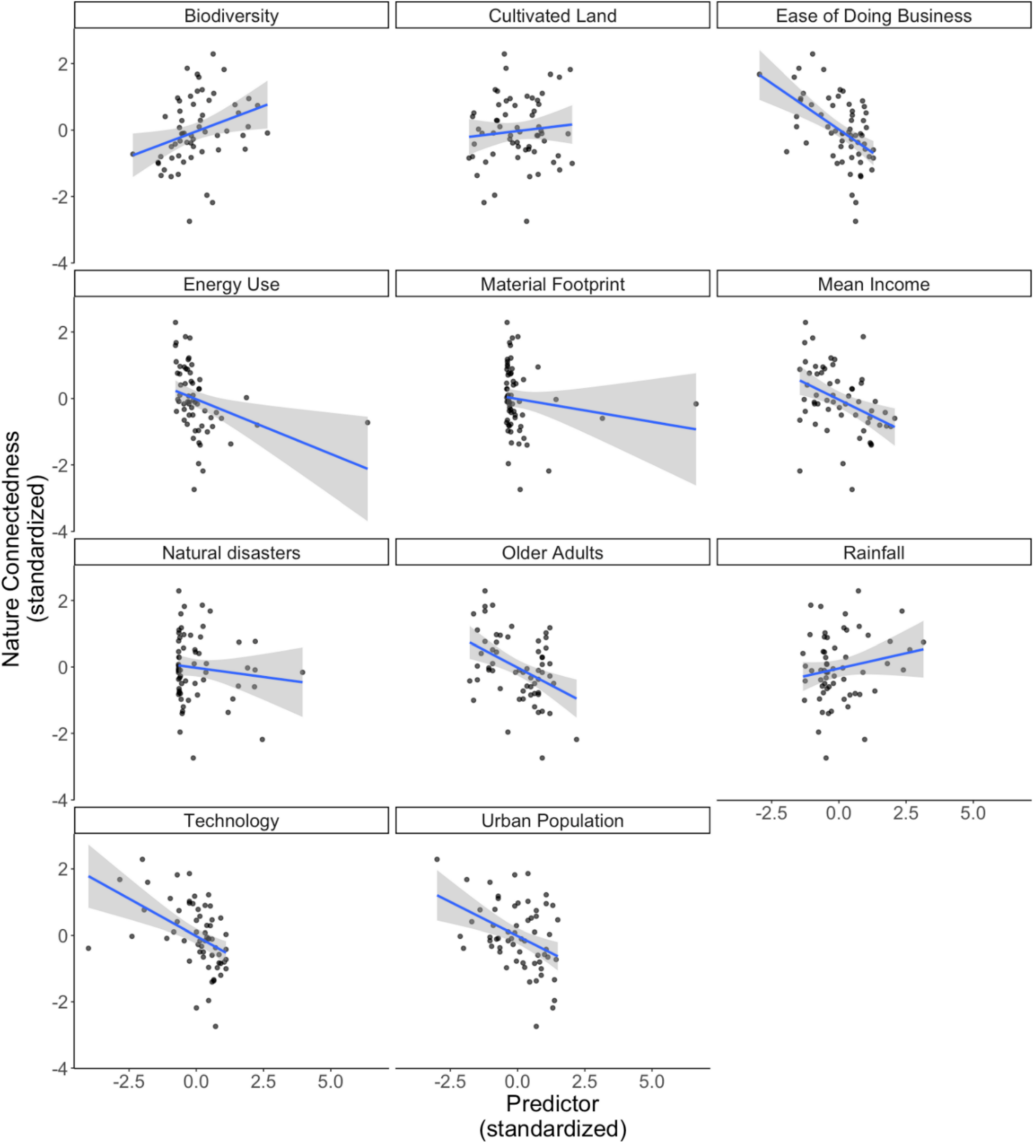
Scatter plots of objective country-level indicators versus nature connectedness. Shaded regions reflect standard-error bands

To examine the relationship between socio-ecological contexts and nature connectedness, we computed simple zero-order correlations between nature connectedness and the metrics outlined in Table 5. Seven of these objective measures demonstrated significant correlations (*p* < 0.05) with nature connectedness (see also Fig. 2). All socio-ecological metrics exhibited a negative correlation, whereas biodiversity showed a positive—albeit the weakest—correlation. The metric for ease of doing business was identified as the strongest correlate.

**Table 5:** Correlation matrix of objective country-level indicators. ^**^Correlation is significant at the 0.01 level (2− tailed). ^*^Correlation is significant at the 0.05 level (2− tailed)

	Nature Connectedness	Urbanisation	Older Adults	Mean Income	Ease of Business	Internet	Energy Use	Biodiversity	Cultivated Land	Material Footprint	Natural disasters	Rainfall	SDG Rank
Nature Connectedness													
Urbanisation	− 0.408^**^												
Older Adults	− 0.419^**^	0.296^*^											
Mean Income	− 0.398^**^	0.456^**^	0.346^**^										
Ease of Business	− 0.537^**^	0.333^**^	0.542^**^	0.555^**^									
Internet	− 0.454^**^	0.703^**^	0.464^**^	0.590^**^	0.679^**^								
Energy Use	− 0.332^**^	0.440^**^	0.171	0.473^**^	0.408^**^	0.465^**^							
Biodiversity	0.304^*^	− 0.144	− 0.299^*^	− 0.317^*^	− 0.282^*^	− 0.384^**^	− 0.455^**^						
Cultivated Land	0.097	− 0.136	− 0.052	− 0.262^*^	− 0.181	− 0.167	− 0.293^*^	− 0.035					
Material Footprint	− 0.136	0.014	0.050	− 0.012	0.157	− 0.075	− 0.015	0.306^*^	0.086				
Natural disasters	− 0.111	− 0.055	− 0.112	− 0.119	− 0.018	− 0.293^*^	− 0.094	0.563^**^	− 0.139	0.700^**^			
Rainfall	0.183	− 0.149	− 0.107	− 0.088	− 0.161	− 0.276^*^	− 0.010	0.513^**^	− 0.195	− 0.037	0.302^*^		
SDG Rank	− 0.399^**^	0.347^**^	0.876^**^	0.521^**^	0.634^**^	0.639^**^	0.275^*^	− 0.363^**^	− 0.149	− 0.049	− 0.206	− 0.062

### Linear analysis using objective indicators

A linear analysis was conducted to predict nature connectedness from variables including Biodiversity, Older Adults, Urban Population, Ease of Business, Internet, Energy Use, and Mean Income. Variables with non-significant correlations were excluded. Multicollinearity was assessed using a bivariate correlation matrix and variance inflation factor (VIF) values (with VIF values > 5.0 typically considered to be an indication of multicollinearity). All inter-correlations were below 0.80, reducing the risk of multicollinearity (Shrestha 2020), and diagnostics suggested acceptable VIF levels (< 3.8). The Durbin-Watson statistic (2.154) was used to check for autocorrelation among the residuals, with values between 1 and 3 generally considered acceptable, and this result indicated no significant concerns. The assumptions of the linear model were checked and met: a P-P plot and histogram of the standardized residuals indicated normality, and a scatterplot of standardized residuals against predicted values showed no discernible pattern, confirming homoscedasticity. The model explained 30% of the variance in nature connectedness, *F*(7, 56) = 4.41, *p* < 0.001, Adj*. R *^2^ = 0.30. Higher levels of nature connectedness were significantly associated with lower Ease of Business scores (β = − 0.405, *p* = 0.030) and lower Urban population (β = − 0.356, *p* = 0.045), after accounting for the other variables.

### Ancillary objective: Comparison of nature connectedness and SDG Scores

The score for progress across the 21 sustainable development goals (SDGs) showed a significant, negative correlation with nature connectedness (*r* = − 0.399) and biodiversity (*r* = − 0.363), with the strongest correlates of SDG scores being Older Adults (*r* = 0.876), Mean Income (*r* = 0.521), Ease of Business (*r* = 0.634), and Internet Usage (*r* = 0.639). A linear analysis predicting SDG ranking scores from Ease of Business, Internet, Older Adults, and Mean Income accounted for 84% of the variance, *F*(4, 57) = 77.30, *p* < 0.001, Adj. *R*^2^ = 0.84. Importantly, SDG scores’ strongest positive correlates are all negative correlates of nature connectedness. This suggests that SDG rank might be more aligned with socioeconomic progress than it is with sustainability, the state of nature, and human relationships with it.

*(RQ3) Which subjective country-level indicators of socio-political values are associated with nature connectedness?*

To investigate the relationship between values and nature connectedness, we performed zero-order correlations between nature connectedness and metrics from the World Values Survey (WVS; Table 6). Due to high correlations between the importance of God, religion, and belief in heaven (approximately 0.9), these three items were combined into a single spirituality construct. The WVS items Science versus Faith (*n* = 37) and Society needs Change (*n* = 37) were available for fewer nations, reducing the number of cases for subsequent analysis but remaining sufficient for linear analysis (Austin and Steyerberg 2015).

**Table 6:** Values correlation matrix (Significant correlates of nature connectedness only)

	Nature Connectedness	Growth over Environment	Importance of Democracy	Friends Importance in Life	Science versus Faith	Society needs radical change	Society needs gradual change	Spirituality	Left versus Right
Nature Connectedness									
Growth over Environment	0.308^*^								
Importance of Democracy	− 0.340^*^	− 0.437^**^							
Friends Importance in Life	− 0.294^*^	− 0.126	0.237						
Science versus Faith	0.543^**^	0.328	− 0.308	− 0.260					
Society needs radical change	0.387^*^	0.666^**^	− 0.580^**^	0.129	0.271				
Society needs gradual change	− 0.375^*^	− 0.148	0.324	0.099	− 0.151	− 0.399^*^			
Spirituality	0.480^**^	0.460^**^	− 0.290^*^	− 0.140	0.245	0.552^**^	− 0.289		
Left versus Right	0.411**	0.384**	− 0.315*	− 0.450**	0.348	0.353	− 0.405*	0.651**	
SDG Rank	− 0.399**	− 0.454**	0.417**	0.097	− 0.527**	− 0.528**	0.243	− 0.793**	− 647**

**Fig. 3:**
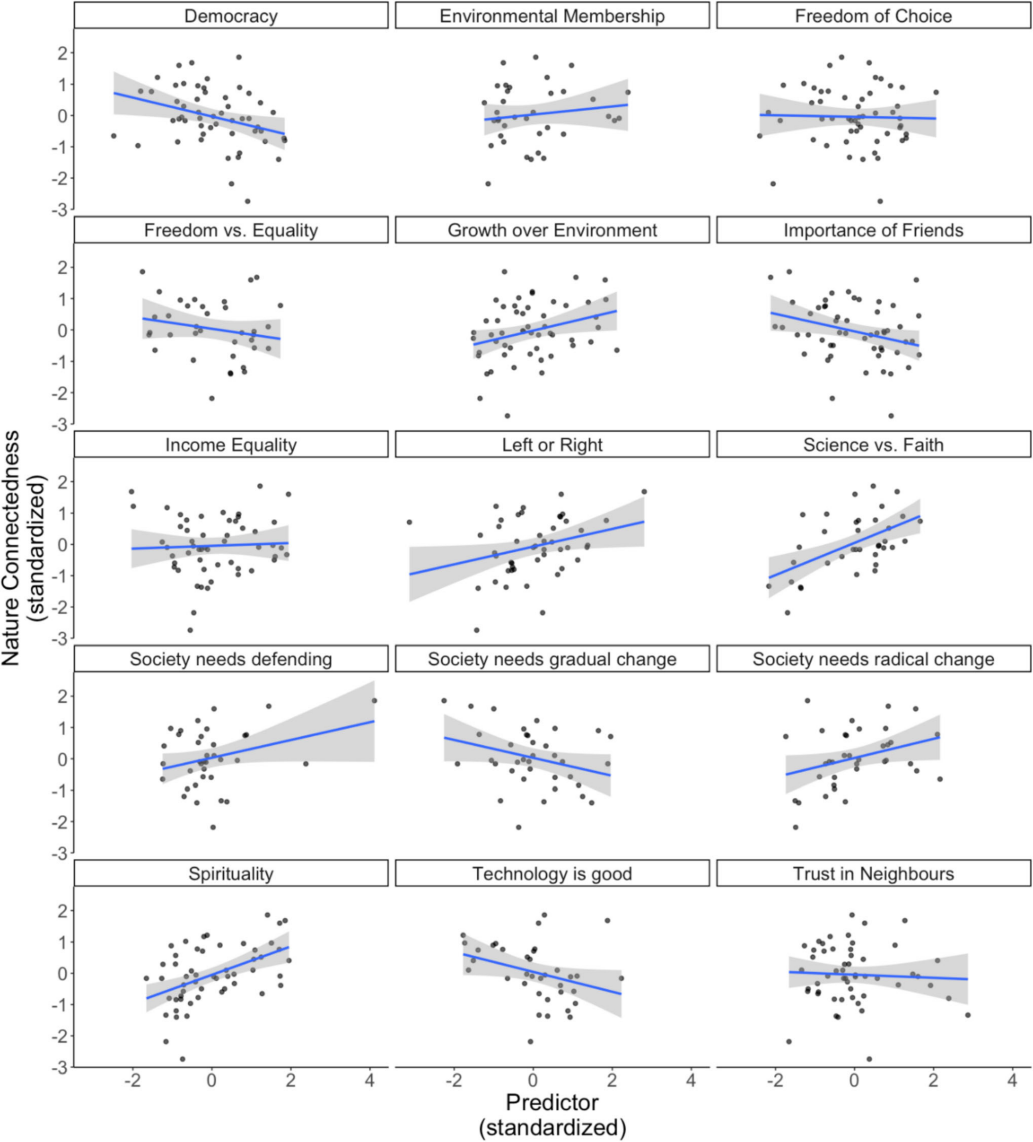
Scatter plots of socio-political values versus nature connectedness. Shaded regions reflect standard-error bands

Regarding the value measures, nature connectedness showed the highest correlations with Spirituality and Science versus Faith. There were moderate, positive correlations with Left versus Right, Growth over Environment, and Society needs Radical Change. Moderate, negative correlations were observed for the Importance of Democracy, Friends, and Society needs Gradual Change (see Table 6 and Fig. 3).

**Fig. 4:**
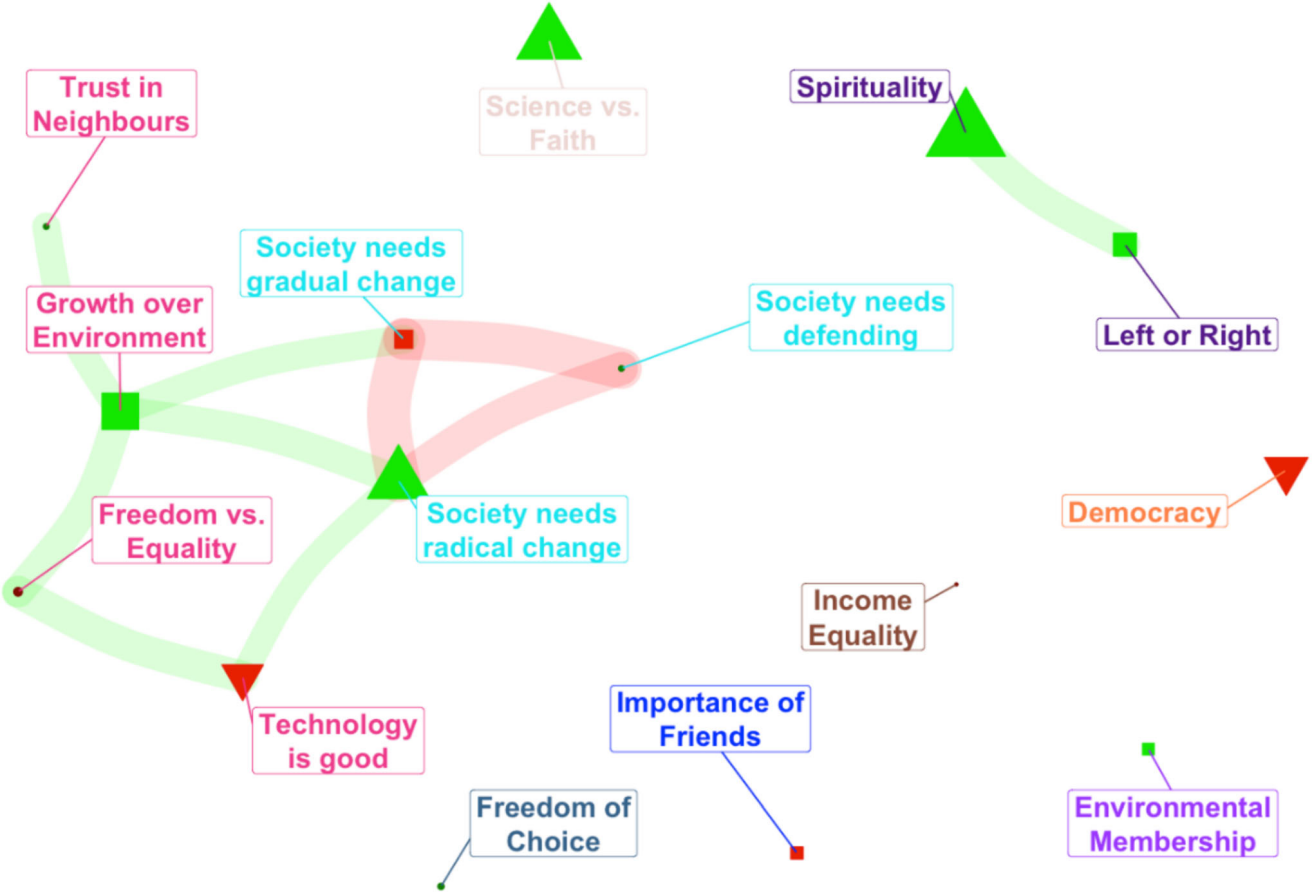
Network analysis of socio-political values and their relationship to nature connectedness (Red nodes indicate a negative correlation with nature connectedness, as shown in the correlation matrix. Green nodes denote a positive correlation with nature connectedness. Triangles represent correlations in the top third for their colour group; squares for the middle third; circles for the bottom third. Red bands indicate negative partial correlations, while green bands indicate positive partial correlations)

In order to visualise inter-relationships, we conducted a network analysis (see Fig. 4). The clusters show that both Science versus Faith and spirituality are largely independent in the network, not being well connected to the primary cluster. Further, both were independent of each other, suggesting that there is an important distinction between general spirituality and the specific cultural tension between science and faith. Additionally, the clustering suggests that technology, growth orientation, and freedom all form a cluster which was distinct but related to a cluster related to broad beliefs about the state of society.

### Linear analysis using values indicators

We ran a linear analysis predicting nature connectedness from Growth over Environment, Importance of Democracy, Importance of Friends, Spirituality, Science versus Faith, and need for radical or gradual Societal Change. We did not include variables with non-significant correlations. Multicollinearity was assessed using a bivariate correlation matrix and VIF values. All inter-correlations were below 0.80, reducing the risk of multicollinearity (Shrestha 2020), and diagnostics suggested acceptable levels (VIFs < 4.9). The Durbin-Watson statistic (1.327) was used to check for autocorrelation among the residuals, and this result indicated no significant concerns. The assumptions of the linear model were checked. While a slight deviation from perfect normality was observed in the P-P plot, the histogram of the standardized residuals was approximately bell-shaped, and a scatterplot of standardized residuals against predicted values confirmed homoscedasticity with no discernible pattern, indicating the model’s assumptions were sufficiently met for this sample size. The model accounted for 68% of the variance in nature connectedness,* F*(8, 28) = 8.68, *p* < 0.001, Adj. *R*^2^ = 0.69. Higher levels of nature connectedness were only significantly associated with higher Spirituality (β = 0.813, *p* = 0.001) and higher Science versus Faith (β = 0.405, *p* = 0.005) after accounting for the other variables.

### Development of conceptual model

The analysis of economic, socio-technical, and environmental indicators was based on the framework proposed by Richardson et al. (2022). While the Kaiser criterion and factor analysis supported a three-factor structure, Velicer’s MAP test suggested a single underlying component. However, the primary objective of this analysis was not simply data-driven item-reduction, but the theory-informed development of a meaningful, interpretable framework for understanding the macro-factors influencing the human–nature relationship. While the MAP test’s single-component result confirms that all indicators are part of a larger, single-dimension construct (e.g. ‘all macro-factors that influence nature connectedness’), this finding and opportunity to minimise dimensionality would obscure meaningful distinctions between socioeconomic, environmental, and land use dynamics—each of which has distinct implications for nature connectedness and intervention design. Merging a diverse set of indicators into a single, undifferentiated group, while statistically plausible, would obscure critical distinctions between social, economic, and environmental factors, making it difficult to identify actionable insights for policy interventions or conduct nuanced research. Thus, it is argued that the significance of items derived from linear analysis informed by insights from the network analysis presents a better approach for the development of a tentative conceptual model.

The 3-factor solution detailed above offers a more coherent and theoretically sound interpretation than strict adherence to a single statistical test and aligns with established disciplinary distinctions. The first factor, socioeconomic (indicators related to consumption, commerce, and ‘extinction of experience’), reflects a coherent socioeconomic dimension of the human–nature relationship. The network analysis results, which clustered Older Adults, Ease of Business, Technology, and Urban Population, further support this finding, providing a converging line of evidence from a complementary analytical method. The second factor, environment, groups indicators that are all broadly environmental, providing another distinct and interpretable dimension. The third factor, weather and land use, cleanly separates rainfall and cultivated land, two factors that are distinct from the other two groups and are crucial for understanding ecological dynamics.

The findings indicate that significant relationships between objective country-level indicators of socio-ecological context and nature connectedness are primarily urbanisation and ease of business, with biodiversity highlighted in the network analysis. Urbanisation, or non-rural living away from nature, clearly links to biodiversity and access to nature. Regarding values, they accounted for a substantially larger proportion of the variance in nature connectedness; however, identifying key factors with the same depth of analysis was not feasible. Nonetheless, some distinct directions emerged from linear analysis and network analysis. These analyses suggested that spiritual beliefs and attitudes towards science and technology are distinct and crucial correlates of nature connectedness. In sum, the key macro-level influences that interact to shape levels of nature connectedness are ease of business, urbanisation, and access to biodiversity from the socio-ecological context. With spirituality and the balance between dependence on faith or science from the socio-political values.

**Fig. 5:**
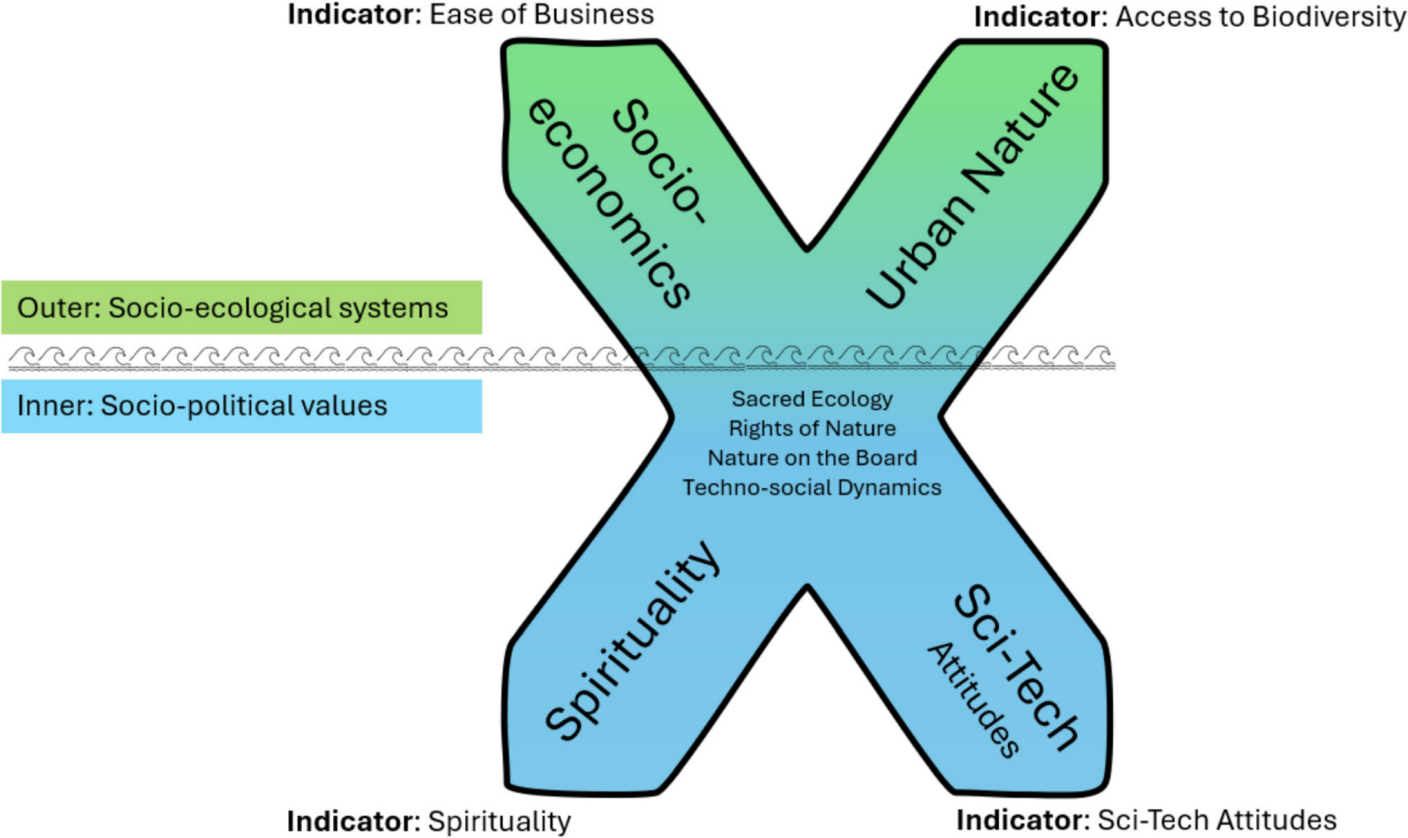
How to target the human–nature relationship: key macro-factors in the human–nature relationship and approaches to change

These four factors from the analysis are positioned within a tentative conceptual model represented by an ‘X’ (see Fig. 5). Rather than a conceptual model driven by World Bank terminology, for the purposes of interpretation and future work on the human–nature relationship, each of the four arms has a suggested name: Urban Nature reflects the meaning of urbanisation in the current context; Sci-tech Attitudes reflect the balance between dependence on faith or science and sits opposite Spirituality, which reflects humanity’s search for deeper meaning and connection beyond the material world. Finally, for ease of business activity to be a significant factor in nature connectedness it must reflect the relationship between business, societal structures, policies, and societal impact, reflected by the term socioeconomics.

### Ancillary objective: Comparison of nature connectedness and SDG rank scores

Table 6 shows that Sustainable Development Rank Scores showed a high negative correlation with Spirituality (*r* = − 0.793). There were also moderate, negative relationships with Science versus Faith, Society needs Radical Change, and Growth over Environment. Finally, there was a moderate correlation with the Importance of Democracy. SDG scores tended to be higher in stable countries with positive attitudes to science where democracy is seen as more important than spirituality.

## Discussion

Nature connectedness is increasingly recognised as a critical factor in addressing environmental crises and as a construct that can be targeted for transformative change (IPBES 2024). However, there is limited understanding of the macro-level factors influencing the human–nature relationship at a societal level. This study utilised data from a diverse set of 61 countries to explore the relationship between both objective country-level indicators of the socio-ecological context and subjective country-level indicators of socio-political values with nature connectedness. The analysis revealed that several key objective and subjective country-level indicators were significantly associated with nature connectedness. These findings are discussed below, and their groupings are used to propose an updated conceptual model of key macro-factors in the human–nature relationship. Finally, the results are examined in relation to the Sustainable Development Index, emphasising the need for suitable metrics to guide and monitor the human–nature relationship.

### Which objective country-level indicators of socio-ecological context are associated with nature connectedness?

The linear analysis showed that the socio-ecological variables accounted for over 29% of the variance in nature connectedness across the 61 countries, with higher levels of urban population and ease of business showing significant negative relationships. Notably, ease of business was identified as a key factor in the network analysis. These themes of urban population disconnection and business align with the target areas recommended in the IPBES (2024) Transformative Change Report. It is also important to consider the inter-relationships between the various macro-factors. The network analysis depicted in Fig. 2 demonstrated that ease of business clusters with technology, urbanicity, and population metrics, rather than mean income, which was isolated. This clustering of the four strongest negative correlates of nature connectedness suggests that socio-technological development plays a crucial role in the human–nature relationship, rather than wealth. Lastly, although there are stronger correlates, the network analysis also indicated that biodiversity had a strong, positive relationship with nature connectedness.

The location of causality is an important consideration. The existing literature (e.g. Eckersley 2000; Hamilton 2002) suggests that greater socioeconomic progress may be associated with disconnection from nature. Conversely, it could be argued that populations living in harmony with nature are less focused on socioeconomic advancement, indicating that nature connectedness might precede economic development. While causality is a concern, socio-technical–economic systems and structures clearly play a significant role in the human–nature relationship and suggest that further research on the influence of macro-level policy levers is warranted. On a practical level, factors such as technology, business focus, and urbanisation offer potential levers for change. Urbanisation, in particular, is linked to exposure to nature and has a relationship with biodiversity, aligning with the goal of biodiversity restoration.

### Which subjective country-level indicators and socio-political values are associated with nature connectedness?

Moving to indicators from the World Values Survey, the linear analysis showed that socio-political values accounted for over 68% of the variation in nature connectedness, with greater spirituality and feeling that there is too much dependence on science over faith significantly associated with higher levels of nature connectedness. Surprisingly, there was a very weak correlation between nature connectedness and membership of environmental organisations. Similarly, freedom of choice, freedom versus equality, trust in neighbours, and valuing for more technology were all weak correlates.

Overall, political culture had few significant associations, beyond a limited role for left or right leaning, which was clustered with spirituality in the network analysis. Views on the dominance of science or faith had a strong, independent influence. The network analysis showed that most of the remaining values related to democracy, society, freedom, trust, equality, politics, technological progress, had many inter-relationships. These form two clusters that can be termed societal cooperation and collective worldviews. This again highlights the unexpectedly isolated and less significant role of environmental organisation membership. As one would expect, values matter, yet the environmental values one might have expected to be important instead appear less critical than those related to spiritualty, faith, and science. The network analysis also showed a positive association between valuing growth over the environment and the need for radical, rather than gradual change. This, together with the negative relationships between nature connectedness and valuing social connections, stable society, environmental protection, and democracy likely reflect disparities in affluence between countries, often summarised by the grouping of global North and South. Where affluence is low, growth is seen as more important than the environment, even though nature connectedness is higher.

The results highlight values related to faith and spirituality, which is also highlighted in the IPBES (2024) analysis of aspirations for desirable futures for humans and nature. Ultimately, spirituality relates to the human desire to find meaning in life and a more spiritual outlook can be part of a connection with nature (Niigaaniin and MacNeill 2022). This link provides some practical ways forward to improve the human–nature relationship. For example, the pathways to nature connectedness (Lumber et al. 2017) include meaning—and incidentally, experimental manipulations of reflections on meaning in life have shown that meaning is causally associated with increased nature connectedness (Lengieza 2024). Moreover, there are proposals, supported by authoritative evidence reviews (e.g. IPBES 2024), on how this can be operationalised at societal scale to increase sensory, meaningful and emotional engagement with nature across policy areas such as education, health, housing, arts, health and transport (Richardson et al. 2022).

### How well do the previously derived socio-ecological context indicator groupings fit the categories derived by Richardson et al’s (2022) typology?

The analysis of economic, socio-technical, and environmental indicators was based on the framework proposed by Richardson et al. (2022). The current study, however, identifies limitations in that initial framework due to the combination of experience extinction and consumption and commerce items in the factor analysis, which suggested six broadly socioeconomic items in a socioeconomic factor. The ease of business metric reflects this group and the original indicator group of commerce effectively. This grouping also includes urbanisation and older adults from the extinction of experience group. Therefore, the analysis suggests merging these groups, as described in the development of the tentative conceptual model.

### Tentative conceptual model

Recognising the failing human–nature relationship as a causal issue of climate change and biodiversity loss, there is a need for policy initiatives to transform this relationship (IPBES 2024). Evidence-based frameworks, such as the pathways to nature connectedness, are informing policy and practice (Richardson et al. 2020; SEI and CEEW 2022). However, targeting societal leverage points, such as macro-factors, is necessary. Since the initial framework proposed by Richardson et al. (2022) for exploring macro-factors in nature connectedness did not align with the results, it was necessary to propose a new conceptual model, see Fig. 4. Such a model will be useful for informing future research and policy initiatives. To facilitate such work, each factor arm in the proposed model has a suggested indicator name: Access to Biodiversity for Urban Nature and Sci-tech Attitudes for the balance between Spirituality and Science, with Ease of Business and Spiritualty remaining the same. Indicators and metrics matter as policy-driven action tends to follow the metrics by which success is operationalised. A relevant recent example is the Kunming-Montreal Global Biodiversity Framework (Convention on Biological Diversity 2022), which recognises that nature in urban and densely populated societies can influence nature connectedness (Target 12). However, the monitoring indicator selected is solely access based, namely the ‘Average share of the built-up area of cities that is green/blue space for public use for all.’ In the spirit of the Iceberg model (Evbuoma et al. 2021), the socio-ecological pair are positioned above the socio-political values pair. The socio-ecological context of business systems and urbanisation represent tangible parts of the ‘outer’ world ‘above the waves,’ while factors derived from values such as spirituality and attitudes towards science reflect the ‘inner’ world of the human mind. Figure 5 presents the key macro-factors from the results above in a way that acknowledges the interaction and integration between the inner and outer domains suggested by embodied and situated approaches to mind (c.f. Thompson 2010). This emphasises the need for policy and research to advance beyond tangible features and infrastructure to encompass faith and values—and cultural factors, more broadly. This is particularly significant as values explained a greater proportion of the variance in nature connectedness, as indicated by the blue shading.

The suggested axis pairings are posited rather than derived directly from the results. Attitudes to technology, as measured by internet usage in the current study, are positively tied to socioeconomic development. This axis relates to techno-social dynamics. Similarly, broadly in the human–nature relationship context, spiritual aspects are related to higher levels of biodiversity and lower urbanisation, suggesting a form of sacred ecology or faith in nature, which in policy terms relates to urban green spaces.

### Future research

The tentative conceptual model of key macro-factors in the human–nature relationship and approaches to change has a basis in the results, but also forms a catalyst for discussion on the direction of future research. The upper socio-ecological systems indicate a need for changes in political, economic, and business regulations related to urbanisation and biodiversity in order to improve the human–nature relationship, reflecting the current trend in eco-social policy research (Schulze-Waltrup 2023). These changes could range from concepts such as Biodiversity Net Gain in the UK to initiatives such as rights for nature (Borràs 2016) and nature being represented on company boards of directors (Faith in Nature 2022). Structural changes have significant transformative potential (Eckersley 2022), and theories of systems change (e.g. leverage points; Meadows 1999) have been integrated with the pathways to nature connectedness framework (Richardson et al. 2020). Moreover, governance systems should recognise the interaction between structures and worldviews (techno-social dynamics). These socio-ecological systems also encompass initiatives to introduce biodiversity into urban areas.

The concept of structural socio-ecological systems and the interconnected and dynamic relationship between human societies and their surrounding ecological environments represented in the upper half of the ‘X’ are more familiar and tangible than notions of ‘techno-spiritual’ integration suggested by the lower half, and the WVS faith versus science item itself. The rapid technological evolution since the industrial revolution, which coincided with environmental crises and a human–nature disconnect, has not been matched by rapid spiritual development. This disparity raises questions regarding how a technologically advanced culture accommodates spirituality (cf. Coeckelbergh 2010).

Similarly, notions of ‘sacred green spaces’ that encompass spirituality and access to biodiversity are less familiar. The associations found between spiritual beliefs and biodiversity for fostering nature connectedness suggests a sacred ecology informed by knowledge–practice–belief systems surrounding the relationships between living beings and their environment (Berkes 2017; Burgos-Ayala et al. 2020). Although sacredness, spirituality, and the human–nature relationship are challenging areas for policy formulation, they are addressed in research (e.g. Ives and Kidwell 2019), proposed in UK policy (Dasgupta 2021), and implemented in certain countries. For instance, Ecuador has incorporated the rights of nature and the Indigenous concept of *Buen Vivir*, or living in harmony, into its constitution (Altmann 2020). Concepts such as sacredness and harmony are fundamental to fostering a positive relationship with nature, as exemplified by the principle of *Buen Vivir*. Achieving harmonious societies necessitates a bio-scientific perspective that integrates science, spirituality, commerce, and biodiversity (cf. Ibrahim et al. 2018), promoting a relational approach that avoids positioning business against nature and spirit against science. The current analysis indicates that there are metrics and indicators available that could assist in shaping change and monitoring progress.

While policy proposals can be informed by the current analysis, initiatives such as *Buen Vivir* highlight the need to consider the context of specific countries and communities. For instance, in more affluent countries, there may be a greater need to balance scientific advancement with spirituality, the adoption of sustainable business practices, and the protection and restoration of biodiversity. Conversely, in less affluent countries, the emphasis might be on enhancing science and technology due to their crucial role in fostering economic development (Ahmed and Shimada 2019). However, it is imperative that this technological advancement be pursued alongside efforts to preserve biodiversity and spirituality, as well as implementing thoughtful business regulations.

The discussion of factors involved in the human–nature relationship engages with a variety of literatures from systems and structural changes related to economic and political environments and biodiversity. However, it does not constitute a focussed field of study, despite its importance for achieving sustainability. There is a need to synthesise perspectives to examine the individual, cultural, and spatial factors influencing people’s relationships with nature and the complex interactions within societal systems.

### How does nature connectedness compare as an indicator to SDG Ranking?

SDG ranking scores were very highly correlated to lower Spirituality and an Older Population with strong relationships to income, ease of business, and internet usage. SDG scores tend to be higher in stable countries with positive attitudes to science and where democracy is valued. SDG scores could reflect that developed countries are doing more because they are less sustainable, but we suggest they reflect level of development with much of the variance explained by socioeconomic indicators. The SDGs are more related to socioeconomic development (e.g. poverty, hunger, health, education, industry, justice, clean water and energy), with only two goals related directly to nature: life on land and life on water. The SDGs should tap into or improve the human–nature relationship. Indeed, only one SDG has a significant correlation to nature connectedness and that is at a moderate level (Feucht et al. 2024). These findings, together with those above, highlight the potential of initiatives such as the Inner Development Goals which aim to promote inner growth through purposeful and productive lives and bring about transformational change in sustainability (Ankrah et al. 2023).

### Limitations

As discussed in the approach to analysis, although aggregated data provide an initial step to generate population-level hypotheses, it is crucial not to assume that statistical relationships at the group level apply to individuals. As further discussed in the approach, although having data from 61 countries is notable, this is less than a third of all countries worldwide. This figure limits analytic options, but those taken were justifiable. A larger dataset would be more suitable for curvilinear analysis, representing a limitation of the current work. As with any archival data analysis, and especially with archival data analysis at the country level, the indicators used to operationalise these constructs were far from perfect, as those gathered and shared by organisations such as the World Bank tend to focus on commerce, development, and environment. However, overall, analyses within this limited sample of countries revealed that each indicator grouping was associated with country-level nature connectedness. This supports the notion that macro-level factors in these groupings may be related to people’s relationship with nature and provides a list of potential metrics for further investigation with a larger, more globally diverse sample. The sample required translations into several languages, and this presents a potential source of variability, but previous analyses has shown the CNS-7 achieved scalar invariance across gender and age, as well as partial scalar invariance across national groups and languages (Swami et al. 2024). Further, whereas the WVS uses representative samples, the BINS did not, but steps to minimise response biases were taken (Swami et al. 2022). Further still, there is limited understanding of how the two sets of subjective and objective metrics are related to each other. Finally, no items were specifically designed to test Kellert’s framework, so these proxies may not be the best ones. However, Kellert’s framework was an initial starting point to inform exploratory work into macro-factors, the present paper uses it as a stepping-stone to suggest a tentative and hopefully more appropriate conceptual model, rather than test a framework derived for other purposes.

## Conclusion

Our extensive analysis involving numerous countries has identified important macro socio-ecological factors and values indicators that can explain the various levels of nature connectedness. These insights are important for policy initiatives aimed at enhancing the human–nature relationship. The breadth of the study has significant implications for human–nature relationship research, demonstrating that social, scientific, and spiritual values explain nature connectedness more than tangible socio-ecological factors. The results also highlight limitations in the SDG approach and can therefore, help guide global policies to foster a better human–nature relationship. Key findings suggest that socioeconomic conditions, biodiversity, spirituality, and attitudes towards technology are associated with nature connectedness. For a renewed relationship with nature, structural socioeconomic systems and biodiversity efforts must align with ‘techno-spiritual’ aspects. The integration of technological advancements and spiritual evolution is perhaps key for addressing the human–nature disconnect, emphasising the need for new concepts like ‘sacred ecology’ and techno-social dynamics. Addressing these factors is often overlooked in approaches to ensuring the wellbeing of both humanity and the environment.

## Data Availability

The datasets used in this research are publicly available from the sources indicated in the manuscript. Our *R* codes are available upon reasonable request.
