# Publisher Correction: Macro-level determinants of nature connectedness: An exploratory analysis of 61 countries

**DOI:** 10.1007/s13280-025-02342-2

**Published:** 2026-01-21

**Authors:** Miles Richardson, Michael Lengieza, Mathew P. White, Ulrich S. Tran, Martin Voracek, Stefan Stieger, Viren Swami

**Affiliations:** 1 School of Psychology, University of Derby, Kedleston Road, Derby, DE22 1GB UK; 2 Department of Psychology, Durham University, South Road, Durham, DH1 3LE UK; 3 Department of Clinical and Health Psychology, Faculty of Psychology, University of Vienna, Kolingasse 14/16, 1090 Vienna, Austria; 4 Department of Cognition, Emotion, and Methods in Psychology, Faculty of Psychology, University of Vienna, Vienna, Austria; 5 Department of Cognition, Emotion, and Methods in Psychology, Faculty of Psychology, University of Vienna, Vienna, Austria; 6 Department of Psychology and Psychodynamics, Karl Landsteiner University of Health Sciences, Krems an Der Donau, Austria; 7 School of Psychology, Sport, and Sensory Sciences, Anglia Ruskin University, Cambridge, UK; 8 Centre for Psychological Medicine, Perdana University, Kuala Lumpur, Malaysia

**Correction to Ambio (2026) 55:80–100**. 10.1007/s13280-025-02275-w

In this article, the sentence "Conversely, are recognised as one of three major underlying causes of biodiversity loss (IPBES 2024)." appearing in **Introduction** section should be "Conversely, lower levels of nature connectedness are recognised as one of three major underlying causes of biodiversity loss (IPBES 2024).”

